# QTL Identification for Stem Fiber, Strength and Rot Resistance in a DH Population from an Alien Introgression of *Brassica napus*

**DOI:** 10.3390/plants11030373

**Published:** 2022-01-29

**Authors:** Yujiao Shao, Yusen Shen, Feifei He, Zaiyun Li

**Affiliations:** 1 College of Chemistry and Life Science, Hubei University of Education, Wuhan 430070, China; syjsyj520@126.com; 2 Institute of Vegetables, Zhejiang Academy of Agricultural Sciences, Hangzhou 310021, China; 3 Department of Natural Sciences, Shantou Polytechnic, Shantou 515078, China; ffhe@stpt.edu.cn; 4 College of Plant Science and Technology, Huazhong Agricultural University, Wuhan 430070, China; lizaiyun@mail.hzau.edu.cn

**Keywords:** *Brassica napus*, lignin, stem strength, *Sclerotinia sclerotiorum*, QTL mapping

## Abstract

Stem fiber, stem strength and stem-rot resistance are important agronomic traits in *Brassica napus*. To understand the molecular mechanism that controls the stem-related traits, we investigated the stem lignin (ADL), cellulose (Cel), hemicellulose (Hem) content, S/G monolignol ratio (SG), stem breaking force (BF), breaking strength (F) and *Sclerotinia sclerotiorum* resistance (SSR). Each trait was significantly positively or negatively correlated with more than three of the other six traits. QTL mapping for ADL, Cel, Hem, SG, BF, F and SSR were performed using a doubled haploid population derived from an intertribal *B. napus* introgression line ‘Y689′ crossed with *B. napus* cv. ‘Westar’. A total of 67 additive QTL were identified and integrated into 55 consensus QTL by meta-analysis. Among the 55 consensus QTL, 23 (41.8%) QTL were co-located and were integrated into 11 unique QTL. The QTL by environment (Q × E) interactions were analyzed and 22 combined QTL were identified. In addition, candidate genes within the QTL intervals were proposed based on the known function of *Arabidopsis* orthologs. These results provided valuable information for improving lodging resistance, *S. sclerotiorum* resistance and mechanized harvesting of *B. napus*.

## 1. Introduction

Rapeseed (*Brassica napus*) is a widely grown crop that is used as vegetable oil for human, biodiesel energy for industry and feeding protein for animals. Fiber-related components, including lignin, cellulose (Cel) and hemicellulose (Hem), have played important roles in stem lodging [1,2], biomass digestibility [3] and *Sclerotinia sclerotiorum* (causing stem rot) resistance (SSR) [4,5] in *B*. *napus*. Fibers are complex traits that are controlled by a number of genes [6]. Lignin is the most important fiber component and is composed of three units: syringyl (S), guaiacyl (G) and p-hydroxyphenyl (H) monolignols. As a dicot, *B*. *napus* primary contains S and G monolignols. Several lignin biosynthesis-related genes have been reported in *Arabidopsis thaliana* [7,8,9,10], rice [11,12], maize [13,14] and some ligneous plants [15,16,17].

In *B. napus*, seed fiber-related traits have been emphasized because of their effect on seed oil content and meal value. Some major QTL or candidate genes associated with seed acid detergent lignin (ADL) content have been identified [6,18,19,20]. For stem fiber-related traits, Wei et al. [2] uncovered eight and nine significant SNPs involved in ADL content and the syringyl/guaiacyl (S/G) monolignol ratio, respectively. Moreover, the S/G monolignol ratio was significantly negatively correlated with lodging resistance and stem disease susceptibility. A recent study proved that decreasing the S/G ratio by knocking out of the lignin pathway gene, *BnF5H*, could improve the *S. sclerotiorum* resistance and increase stem strength [5].

Stem strength has been proposed as a key index of lodging resistance because of its difficulty for scoring lodging resistance under natural field conditions [21,22]. However, stem strength is a complex trait affected by related basal internode traits, such as stem diameter and cell wall components. The correlation between stem strength and cell wall components has not been elucidated in terms of the significance of cellulose or lignin [23]. Some progress of genetic studies on stem strength has been made in wheat [22,24,25], barley [26], rice [27], maize [28,29,30] and soybean [31,32]. In *B. napus*, stem strength has usually been studied along with lodging resistance. Wei et al. [2] decomposed stem strength into breaking force (BF) and breaking strength (F), and detected 11 and 7 significantly associated SNPs, respectively. One gene, *BnaA01g26700D*, encoding a TEOSINTE BRANCHED 1, CYCLOIDEA, PCF1 (TCP) transcription factor (TF), was deemed to be a candidate gene regulating stem strength. Li et al. [21] studied the stem lodging-related traits and identified 35 SNPs significantly associated with stem strength, which contributed to 23.3% of the cumulative phenotypic variation.

*Sclerotinia sclerotiorum* is a necrotrophic lifestyle fungal pathogen, which can cause 0–20% of yield loss every year in *B. napus* and can reach up to 80% in severely infected fields in China [33]. Very recently, some lignin biosynthesis pathway genes were reported to be related to *S. sclerotiorum* resistance in *B. napus*, such as *BnF5H* [5], *BnCAD5* [34] and *BnaC.CCR2* [4]. These findings indicate that increasing the lignin content in the stems of *B. napus* might be an effective strategy for controlling *S. sclerotiorum*.

In the present study, we analyzed the phenotypic correlation of ADL, Cel, Hem, SG, BF, F and SSR, and performed QTL mapping, meta-analysis and QTL by environment interaction analysis to dissect the genetic basis of these traits. Some fiber or disease related genes located within the QTL intervals were intended as candidate genes. These results may pave the way for deciphering the genetic control of stem-related traits in *B. napus.*

## 2. Results

### 2.1. Phenotypic Performances of the Parents and DH Lines

**Figure 1:**
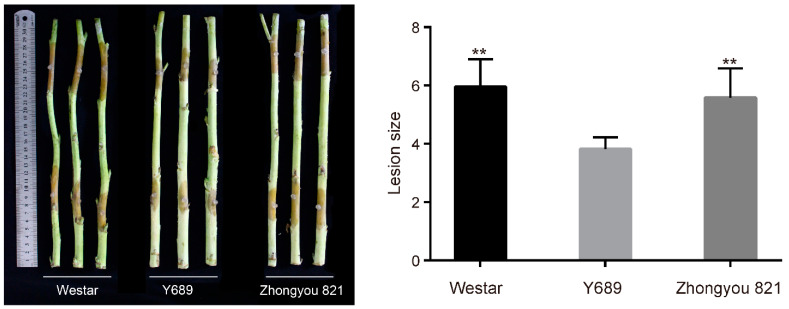
Resistance to *Sclerotinia sclerotiorum* of the two parents ‘Westar’ and ‘Y689′, and the control line ‘Zhongyou 821′. The level of resistance of ‘Y689′ to *S. sclerotiorum* was significant compared to ‘Westar’ and ‘Zhongyou 821′. ** means the significance level between Y689 and the other two lines, *p* ≤ 0.01.

**Figure 2:**
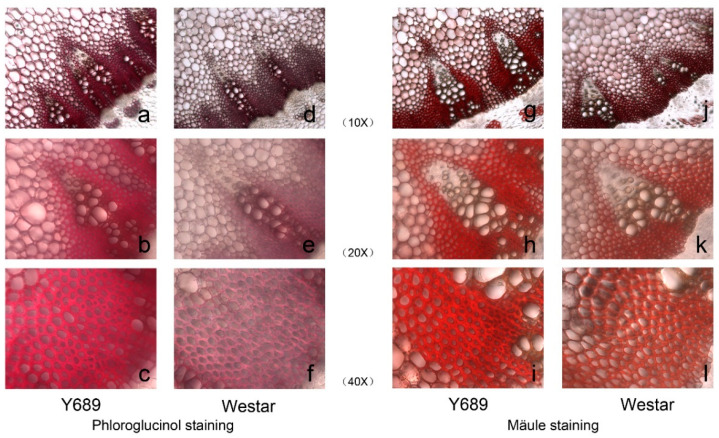
Phloroglucinol and Mäule staining of ‘Y689’ and ‘Westar’. (**a**–**c**) Transverse internode sections of ‘Y689’ after phloroglucinol staining. (**d**–**f**) Transverse internode sections of ‘Westar’ after phloroglucinol staining. The lignin quantity of ‘Y689’ is more than ‘Westar’. (**g**–**i**) Transverse internode sections of ‘Y689’ after Mäule staining. (**j**–**l**) Transverse internode sections of ‘Westar’ after Mäule staining. The Mäule reagent stains: G residues yellow and S residues red. Magnification 10×, 20×, and 30×.

At maturity stage, the detached stems of the two parents ‘Y689’ and ‘Westar’ as well as the control line ‘Zhongyou 821’ were inoculated with *S. sclerotiorum*. As a result, the level of resistance of ‘Y689’ to *S. sclerotiorum* was significant compared to ‘Westar’ and ‘Zhongyou 821’ (Figure 1). We then examined histochemical transverse sections of the second stem internodes of the parents stained with phloroglucinol reagent, the vascular bundle regions were stained dark red in ‘Y689’ but were light red in ‘Westar’ (Figure 2a), indicating the higher lignin quantity of ‘Y689’ than ‘Westar’. The lignin compositions of the parents were also estimated by Mälue staining, with the Mäule reagent stains, G residues yellow and S residues red. Fresh hand-cut internode sections of the two parents were stained red in the vascular bundle regions (Figure 2b), indicating that these regions contained abundant S monomers. This result fit well with the measurement using the near-infrared spectroscopy (NIRS) method (Table 1).

**Figure 3:**
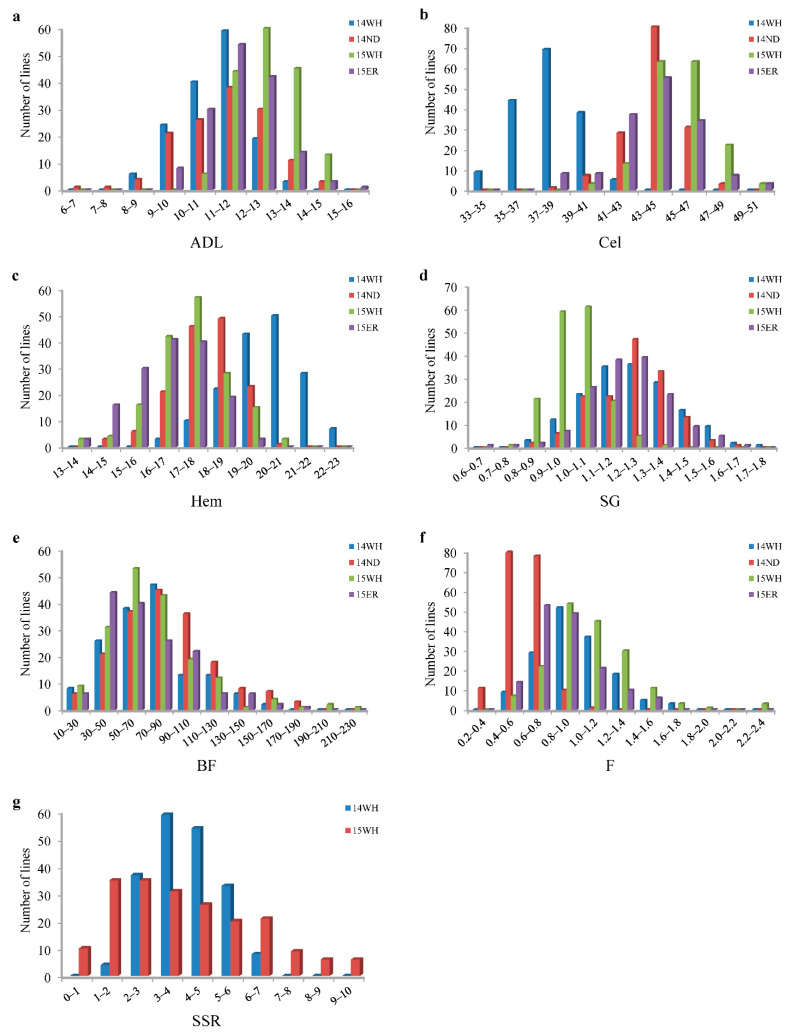
Phenotypic distributions of ADL, Cel, Hem, SG, BF, F and SSR in the YW-DH population derived from the cross ‘Y689′ × ‘Westar’. (**a**) Acid detergent lignin content, (**b**) cellulose content, (**c**) hemicellulose content, (**d**) syringyl/guaiacyl monolignol ratio, (**e**) breaking force, (**f**) breaking strength and (**g**) resistance against *Sclerotinia sclerotiorum*. 14WH, 14ND, 15WH and 15ER represent four environments with different colors. 14WH: Wuhan, 2014–2015; 14ND: Weinan, 2014–2015; 15WH, Wuhan, 2015–2016; 15ER, Ezhou, 2015–2016.

Phenotypic data for ADL, Cel, Hem, SG, BF and F in the DH population were detected over four environments (14WH, 14ND, 15WH and 15ER), and SSR was detected over two environments (14WH and 15WH). The BF, F and SSR of the two parents differed significantly in all experimental environments, whereas for the ADL, Cel, Hem and SG, significant differences (*p* < 0.05) were only detected in two or three environments. Statistical data of the seven traits for the DH population and the parents are listed in Table 1. The DH lines exhibited broad variations among the seven traits. Frequency distributions of the phenotypic values of the seven traits in the DH lines are shown in Figure 3. Among these traits, Cel and Hem differed significantly between the environments of 14WH and the others. SG differed significantly between 15WH and the other environments. F was significantly different between 14ND and the other environments.

The broad-sense heritabilities (*h*^2^) of ADL, Cel, Hem, SG, BF, F and SSR were calculated as 0.66, 0.79, 0.81, 0.56, 0.68, 0.56 and 0.62, respectively (Table 1 and Supplementary Table S1). Within the seven traits, SG and F presented lower heritability, indicating that these two traits were easily influenced by environmental factors. The Pearson’s correlation coefficients between the seven traits ranged from 0.00 to 0.79 (Supplementary Table S2). Each trait was significantly positively or negatively correlated with more than three of the other six traits. For example, Cel was determined to be significantly positively correlated with Hem (*r* = 0.43, *p* < 0.001) and SSR (*r* = 0.32, *p* < 0.001), and negatively correlated with ADL (*r* = −0.16, *p* < 0.05), SG (*r* = −0.16, *p* < 0.001), BF (*r* = −0.33, *p* < 0.001) and F (*r* = −0.39, *p* < 0.001). In these comparisons, BF was highly correlated with F (*r* = 0.79, *p* < 0.001), probably because F was calculated with the following formula: F = BF/(π × (D/2)^2^), where D was the diameter of the stem. Moreover, SSR was significantly positively correlated with Cel (*r* = 0.32, *p* < 0.001) and significantly negatively correlated with BF (−0.32, *p* < 0.001) and F (−0.38, *p* < 0.001), indicating that stems with higher breaking strength/force and lower cellulose content were more resistant against the disease caused by *Sclerotinia sclerotiorum*.

### 2.2. Additive QTL Mapping and Meta-Analysis

**Figure 4:**
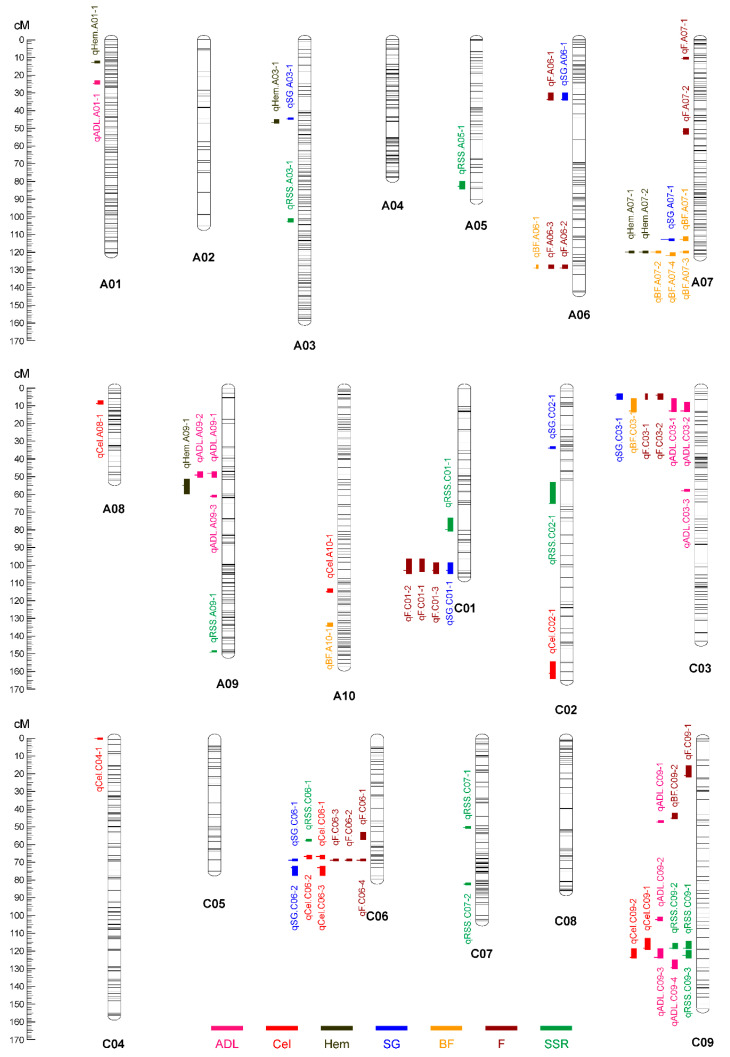
The identified QTL for ADL, Cel, Hem, SG, BF, F and SSR and their chromosome distribution. The length of the vertical bar in the “⊣” symbol indicates the confidence interval of the QTL, and the position of the horizontal bar indicates the peak position of the QTL.

With the method of CIM, a total of 67 significant additive QTL were detected across 2 years (Table 2 and Figure 4). These QTL were primarily distributed on chromosomes C09 (11 QTL), C06 (10 QTL), A07 (9 QTL) and C03 (7 QTL), singly explaining 1.92–27.18% of the phenotypic variation. Further, meta-analysis was used to integrate QTL across different environments (first round) and different traits (second round). In the first round of meta-analysis, 55 consensus QTL were detected, seven of which were major QTL. Notably, these major QTL were mainly clustered on Chromosomes A07 (112.5–120.35 cM) and C09 (116.79–123.5 cM) (Table 2).

For ADL, 11 additive QTL were detected and account for 5.58–16.57% of the phenotypic variation. These QTL were then integrated into nine consensus QTL, with one QTL (cqADL.C09-3) considered to be a major QTL. For Cel, nine additive QTL were detected and accounted for 4.85–12.09% of the phenotypic variation. These QTL were integrated into eight consensus QTL, and no major QTL were detected. For Hem, five additive QTL were detected and accounted for 6.13–12.96% of the phenotypic variation. These QTL were integrated into three consensus QTL, with one QTL (cqHem.A07-1) considered to be a major QTL. For SG, eight additive QTL were detected and accounted for 4.40–27.18% of the phenotypic variation. These QTL were integrated into eight consensus QTL, with one QTL (cqSG.A07-1) found to be a major QTL. For BF and F, 7 and 16 additive QTL were identified, and accounted for 6.25–16.65% and 1.92–12.93% of the phenotypic variation, respectively. The QTL of cqBF.A07-2 was a major QTL for BF, which could be repeatedly detected in the environments of 14WH, 15ER and BLUE, with a mean PVE of 10.43%. The QTL of cqF.C06-2 was a major QTL for F, which could be repeatedly detected in 14WH and 15ER, with a mean PVE of 14.89%. For SSR, 11 additive QTL were detected and accounted for 4.91–17.11% of the phenotypic variation. These QTL were integrated into 10 consensus QTL, with two QTL (cqSSR.C09-1 and cqSSR.C09-2) considered to be major QTL. The QTL of cqSSR.C09-1 could be repeatedly detected in 15WH and BLUE, accounting for 12.64% and 14.26% of the phenotypic variation. The QTL of cqSSR.C09-2 could be detected in 14WH, with the PVE of 17.11%.

In the second round of meta-analysis, 23 of the 55 consensus QTL with overlapping regions were integrated into 11 unique QTL (Table 3). Of these, four unique QTL (uqA06-1, uqC01-1, uqC03-1 and uqC06-1) were pleiotropic for both SG and F, one unique QTL (uqC09-2) was pleiotropic for ADL, Cel and SSR. A high percentage (41.8%) of the consensus QTL detected in different traits could be integrated into unique QTL, which were in accordance with the result that these traits were significantly correlated with one another (Supplementary Table S2).

### 2.3. QTL by Environment Interaction Mapping

Based on the phenotypic and genotypic data, QEI mapping was performed to verify the significantly additive QTL and to detect some probable interaction between additive QTL and the environments. As a result, a total of 22 combined QTL associated with ADL, Cel, Hem, SG, BF, F and SSR were identified, using LOD thresholds of 4.98, 5.07, 4.97, 5.04, 5.00, 4.91 and 3.84, respectively (Table 4). Of these, 15 QTL (68.2%) were also detected by CIM, including five major QTL (IqADL.C09-1, IqHem.A07-1, IqBF.A07-1, IqF.C06-1 and IqSSR.C09-1). With these combined QTL, IqF.C06-1 associated with F presented the strongest QEI effect, with PVE (A) and PVE (A by E) values of 9.61 and 14.15, respectively. Two combined QTL (C07-1 and IqCel.C07-2) associated with Cel were not corresponding major consensus QTL, but exhibited a very strong QEI effect, which could explain 24.29% and 18.45% of the phenotypic variations. Furthermore, values of PVE (A) and PVE (A by E) were compared to evaluate the additive and QEI effect of each combined QTL. Consequently, the PVE (A by E) values in all five combined QTL associated with Hem and BF were larger than PVE (A), probably because Hem and BF were less influenced by environmental factors.

### 2.4. Candidate Genes Mining

According to the reference genome of *B. napus* [35] and the functional annotation of *A. thaliana*, 208 genes were harbored in all consensus QTL intervals corresponding to 142 homologous *A. thaliana* genes. A total of 18, 9, 6, 23, 44, 83 and 25 candidate genes were identified for ADL, Cel, Hem, SG, BF, F and SSR, respectively. For ADL, Cel, Hem and SG, candidate genes were associated with fiber biosynthesis or regulation of the metabolic process, such as *4CL1*, *AtMYB103*, *WRKY13*, *PRX17*, *COMT-like1*, *LAC11*, *GH9A1*, *FEI1* and *BXL2*. For BF and F, candidate genes were mainly associated with plant-type cell wall, such as *EARLI1*, *RP1*, *PGK1*, *GAPC2*, *ASP2*, *TUB5*, *MCTP4*, *ACTIN3* and *LRX1*. For SSR, candidate genes belonged to NBS-LRR-encoding genes (Supplementary Table S3).

## 3. Discussion

The stem-related traits of lignin, cellulose and hemicellulose can enhance the mechanical strength of the plant body, which is conducive to lodging resistance, convenient for mechanized harvesting, and can form barriers to pathogen infection. Moreover, the stem related traits have direct or indirect effects on yield because the plants with rigid stem can reduce lodging and enhance *S. sclerotiorum* resistance. In some reports, lodging has been shown to result in a yield reduction of as much as 46% [36] and *S. sclerotiorum* could cause 0–80% yield loss in *B. napus* [33]. Therefore, it is very important to focus on the stem traits in contemporary *B. napus* breeding.

However, the genetic mechanism and the inner relationship of these traits have yet to be clarified in *B. napus*. Previously, we created an alien ingression line by intertribal hybridization between *B. napus* cv. ‘Zhongyou821′ and *Capsella bursa-pastoris* (L.) Medic (2n = 4x = 32), and named it as ‘Y689′. This ingression line showed wooden stems, tight branch angles, and high resistance to *S. sclerotiorum*. These characters should result from the introgression of the genetic element from *C. bursa-pastoris*, which likely resulted in the longer growth period of ‘Y689′ than the recipient cultivar [37]. To better clarify the genetic basis of these traits, we constructed a DH population derived from the cross of ‘Y689′ with *B. napus* cv. ‘Wester’, which was susceptible to *S. sclerotiorum*. In the present study, we analyzed the relationship between the fiber-related traits (lignin, cellulose, hemicellulose and S/G), stem strength (breaking force and strength) and the resistance to *S. sclerotiorum*. We found that the breaking strength (F) was significantly positively correlated with ADL, and significantly negatively correlated with SG and stem disease susceptibility (Supplementary Table S2), indicating that increasing the ADL content, especially the G proportion, could enhance the stem strength and disease resistance. G monolignol played a vital role in stem strength, which supported the result that decreasing the S/G ratio could improve the *S. sclerotiorum* resistance in *B. napus* and increase stem strength [2,5]. Therefore, we could increase the ADL content and alter ADL composition to improve stem strength, lodging and *S. sclerotiorum* resistance in future breeding programs.

Accurate characterization of the phenotype influenced the results of QTL mapping. For a long time, lack of accurate determination of lignocellulose has limited the progress of genetic analysis in *B. napus*. As a quick assay for lignocellulosic component and property analysis, NIRS has been broadly applied in rice [38], *Miscanthus* samples [39] and *B. napus* [18]. In this study, we applied NIRS to determine the content of lignin, cellulose, hemicellulose and the S/G monolignol ratio. In addition, the breaking force and stem diameter were measured to determine the stem strength. However, the stem diameter sometimes varied from different positions upon the stem. Moreover, stem strength was also affected by the positions of knots. In this study, we investigated the phenotyping data across four environments for stem fiber and strength-related traits, and two environments for *S. sclerotiorum* resistance. The BLUP value was calculated across all environments, which could enhance the accuracy of mapping results as it could give the lowest variance of the estimate linear estimators, and the errors do not need to be normal.

In this study, we performed QTL mapping based on the high-density linkage map and phenotypic data of the seven traits. This linkage map was also used for *B. napus* branch angle and plant architecture analysis in our previous publications [37,40], and many stable QTL were identified, indicating the reliability of this linkage map. Thus far, genetic analysis of stem fiber related traits in *B. napus* is limited to one study using GWAS and transcriptomic analyses [2]. To our knowledge, the present study is the first QTL mapping study for stem fiber and strength-related traits was performed based on a bi-parent population in *B. napus*. We compared the physical position of the two results, and found that only one QTL cqBF.A07-3 (A07:19,528–19,664 kb) associated with BF herein was close to the reported significant SNPs (A07: 20,909–20,962 kb). Other QTL regulating stem fiber and strength identified in this study were novel (Supplementary Table S4). For SSR, Li et al. [41] reviewed the reported QTL and found two conserved QTL regions (A09: 22.5–26.5 Mb and C06: 29.5 to 35.4 Mb). We compared this result and the recent studies [42,43,44] with our result, and found the QTL identified in our result were novel (Supplementary Table S4). The detection of these new loci was largely attributable to the introduction of new gene sources for germplasm innovation via distant hybridizations.

By two-round meta-analysis, all the identified QTL were integrated into consensus QTL and further unique QTL. Many consensus QTL were co-located, and could be integrated into unique QTL, indicating that these QTL had pleiotropic effects or multiple genes that were tightly linked. Fine mapping will be necessary to determine the pleiotropic effects of a single QTL or a tight linkage of two QTL in the same region. These results were supported by the correlation analysis that significant correlations existed between the traits of stem fiber, stem strength and disease resistance (Supplementary Table S2).

QEI mapping was conducted to confirm the consensus QTL and estimate QEI effect. In this study, a total of 22 combined QTL were identified by QEI mapping, and some of the QTL presented a strong QEI effect. Of these, 15 QTL could be detected both by single-environment analysis and QEI mapping, which might be better ones for fine mapping and molecular breeding. For most of the combined QTL, the value of PVE (A by E) was lower than PVE (A), indicating the low QEI effect for most of the QTL. For some combined QTL, such as IqCel.C07-2, IqSG.A07-1 and IqF.C06-2, the value of PVE (A by E) was higher than PVE (A), suggesting the strong QEI effect that was detected.

Candidate genes for each trait were screened based on GO annotations. Some noteworthy candidate genes were identified in overlapping regions of fiber-related (ADL, Cel, Hem and SG) QTL. For instance, *BnaC09g44570* corresponding to *AT1G30230*, encoded a guanine nucleotide exchange factor that played an important role in translation elongation. It was reported that silencing this gene could cause a dwarf phenotype with 38% and 20% reduction in total lignin and crystalline cellulose, respectively. This loss-of-function mutant also had a lower S/G lignin monomer ratio relative to wild type plants [45]. In addition, cqHem.A07-1 and cqBF.A07-2 were major co-located QTL associated with Hem and BF, respectively. In this region, *BnaA07g26180* corresponding to *AT1G02640* was identified as candidate gene, encoding a protein similar to a beta-xylosidase. It was reported that overexpressing this gene could increase enzymatic saccharification efficiency in cultured *Arabidopsis* wood cells [46].

## 4. Materials and Methods

### 4.1. Plant Material and Growth Conditions

The plant material consisted of 208 DH lines developed from F_1_ plants of the cross ‘Y689’ × ‘Westar’ by microspore culture [37,40]. ‘Y689’ was an alien introgression line derived from the intertribal cross between *B*. *napus* cv. ‘Zhongyou 821’ and *Capsella bursa-pastoris* (L.) Medic (2n = 4x = 32) [47]. ‘Y689’ showed rigid stems and high resistance to *Sclerotinia sclerotiorum*. Westar is a *B*. *napus* DH line widely used for the studies of genetic transformation [48] because of its high susceptibility to *Sclerotinia sclerotiorum* [49].

DH lines and the two parents were investigated in four experiments for ADL, Cel, Hem, SG, BF and F, and two experiments for SSR. The materials were planted in a semi-winter rapeseed area, Wuhan of Hubei Province (coded WH), in central China for two years (September–May of 2014–2015 and 2015–2016); Ezhou of Hubei Province (coded ER) for one year (September–May of 2015–2016); and a winter rapeseed area, Weinan of Shaanxi Province (coded ND), in northwest China rapeseed area for one year (September–May of 2014–2015). Year–location combinations were treated as environments, for example, 14WH indicates the experiment was conducted during 2014–2015 at the Wuhan location. Detailed information about the environments can be found in Supplementary Table S5.

The DH lines and the two parents were grown using a randomized complete block design with two replicates. Each plot consisted of 10 plants, with 30 cm between rows and 20 cm within rows. The field management followed the common agricultural practices.

### 4.2. Histochemical Staining

According to the protocol established by [50], the histochemical localization of the accumulated lignin was stained with phloroglucinol reagent. Fresh hand-cut sections from the ‘Y689’ and ‘Westar’ plants were incubated for 10 min in phloroglucinol solution (1% in 70% ethanol), the phloroglucinol was removed and treated with 18% HCl for 5 min, then photographed under a light microscope.

Mäule staining was performed to detect the lignin composition. Sections were treated for 5 min with KMnO_4_, rinsed with water, treated with 10% HCl for 5 min, rinsed again with water and mounted in concentrated NH_4_OH for examination [51].

### 4.3. Phenotypic Evaluations and Statistical Analysis

At maturity, six representative plants from the center of each plot were selected for measurements of ADL, Cel, Hem, SG, BF and F. A length of 30 cm fresh stems were detected to measure the breaking force (BF) and the diameter (*D*), using an electronic Vernier caliper. The breaking strength (*F*) was calculated with the formula: *F* = *BF*/ (π × (D/2)^2^) [2].

Stem samples were then dried in a ventilated oven (65 °C) for about one week until the weight was constant. Based on the Near-infrared (NIR) models developed by Southwest University, Chongqing, China [2], the ADL, Cel, G and S monolignins were measured using NIR (FOSS, NIRS 5000) with the WinISI software.

At maturity, three representative plants from the center of each plot were detached to evaluate *Sclerotinia sclerotiorum* resistance. The *S*. *sclerotiorum* isolate was maintained and cultured on potato dextrose agar (PDA) medium in the dark at 22 °C and 6-mm-diameter mycelia agar plugs punched from the growing margin of a 3-day-old culture of *S*. *sclerotiorum* were used as inoculums [52]. Two 6-mm-diameter mycelia agar plugs were then placed above the 30 cm fresh stems with a 10-cm interval. Lesion sizes were measured after 3 days of inoculation with the infection temperature of 22 °C [53].

The data for each plant were averaged to represent the phenotype of a plot, and data from replicated plots were averaged to represent the phenotype of a DH line. Broad-sense heritability (*h*^2^) and correlations among all traits were analyzed using SAS GLM and CORR packages, respectively [37,40].

### 4.4. QTL Mapping

The population and linkage map utilized in this study were also constructed for *B. napus* branch angle [40] and plant architecture [37] analyses in our previous publications. Briefly, the DH population was genotyped by *B. napus* 60 K SNP array, and 3073 available SNP markers were screened for linkage map construction. This map covered a length of 2242.14 cM and had an average marker interval of 0.73 cM [40].

Based on the genotyping and phenotyping data, QTL analysis was conducted using QTL IciMapping V4.1 [54,55] with the inclusive composite interval mapping (ICIM) method. The ICIM-BIP and ICIM-MET functionalities in this software were used for each experiment independently and for QTL-environment interaction analysis, respectively. The thresholds of the logarithm of the odds (LOD) scores for evaluating the statistical significance of QTL effects were determined using 1000 permutations at 95% confidence level.

QTL identified by the ICIM-BIP functionality were named as additive QTL. Through a two-round strategy of QTL meta-analysis [56], the additive QTL were integrated into consensus QTL for the same trait, and then the consensus QTL were integrated into unique QTL for different traits, using the software BioMercator V4.2 [57]. QTL identified by the ICIM-MET functionality were named as combined QTL.

The QTL nomenclature was based on the description of Shen et al. [40]. Additive QTL was designated with a “q” followed by the abbreviation of trait name, and the order on the chromosomes (e.g., qADL.A01-1). Consensus QTL, unique QTL and combined QTL were thus designated with the initial letters “cq-”, “uq-”, and “Iq-”, (e.g., cqADL.A01-1, uqA06-1 and IqADL.A09-1), respectively.

### 4.5. Candidate Gene Mining

The genetic linkage map could be aligned to the *B. napus* reference genome by BLAST. Based on the collinearity relationship between the genetic linkage map and the reference genome, genes within QTL regions were BLAST to the *A. thaliana* genome. These genes were further annotated with gene ontology (GO) terms, and genes belonging to related GO terms were regarded as potential candidate genes. For ADL and SG, the GO term was GO: 0009808 (lignin metabolic process). For Cel, the GO term was GO: 0030243 (cellulose metabolic process). For Hem, the GO term was GO: 0010410 (hemicellulose metabolic process). For BF and F, the GO term was GO: 0009505 (plant-type cell wall). For SSR, those encoding proteins containing NBS-LRR domains published from [35] were regarded as the most plausible candidate genes.

## 5. Conclusions

The stem lignin, cellulose, hemicellulose, syringyl/guaiacyl monolignol ratio, breaking force, breaking strength and *S. sclerotiorum* resistance were investigated in one DH population consisting of 208 lines in *B. napus*. We found that the breaking strength was significantly positively correlated with stem lignin content and *S. sclerotiorum* resistance, and significantly negatively correlated with the syringyl/guaiacyl monolignol ratio. QTL mapping was performed for these seven traits, and a total of 67 additive QTL were identified. These QTL were integrated into 55 consensus QTL, 23 of which were then integrated into 11 unique QTL by meta-analysis. To confirm the QTL reliability and assess the QTL by environment interaction (QEI) effect, QEI mapping was conducted and 22 combined QTL were identified. In addition, candidate genes within the QTL intervals were proposed based on the known function of *Arabidopsis* orthologs. One gene, *BnaC09g44570*, located in overlapping regions of fiber-related (ADL, Cel, Hem and SG) QTL, likely played an important role for fiber biosynthesis. These results provided valuable information for improving lodging resistance, *S. sclerotiorum* resistance and mechanized harvesting of *B. napus*.

## Figures and Tables

**Table 1:** Statistical analysis of ADL, Cel, Hem, SG, BF, F and SSR for the DH lines and their parents.

Trait	Environment	Parent Y689	Parent Westar ^a^	DH Lines Range	Mean ± SD	Skewness	Kurtosis	Heritability
ADL	14WH	11.68 ± 1.21	10.91 ± 1.53 *	8.31–13.83	10.98 ± 1.07	−0.18	−0.25	0.66
	14ND	12.61 ± 1.23	11.67 ± 2.53	6.66–14.43	11.28 ± 1.42	−0.29	0.17	
	15WH	13.23 ± 1.46	12.55 ± 1.52 **	10.25–14.88	12.62 ± 0.90	0.09	−0.21	
	15ER	12.69 ± 1.32	11.71 ± 1.35 ***	9.10–15.34	11.75 ± 1.08	0.28	0.23	
Cel	14WH	38.49 ± 1.85	36.88 ± 1.23 **	33.37–42.63	37.88 ± 1.71	−0.16	−0.18	0.79
	14ND	46.51 ± 1.64	41.87 ± 1.45	38.98–48.61	43.95 ± 1.65	−0.28	0.39	
	15WH	46.09 ± 1.92	44.02 ± 1.63 *	39.86–49.68	45.20 ± 1.77	−0.10	0.29	
	15ER	45.38 ± 2.03	42.81 ± 2.05	37.55–50.26	43.68 ± 2.34	−0.14	0.27	
Hem	14WH	18.58 ± 1.23	17.07 ± 1.36 **	16.42–23.04	20.00 ± 1.29	−0.21	−0.17	0.81
	14ND	20.38 ± 1.32	19.93 ± 1.23 *	14.10–20.06	17.85 ± 1.11	−0.50	0.34	
	15WH	19.32 ± 1.54	14.71 ± 1.87 ***	13.42–20.69	17.31 ± 1.33	−0.14	0.40	
	15ER	16.57 ± 1.36	15.43 ± 1.65	13.42–19.95	16.62 ± 1.30	−0.06	−0.42	
SG	14WH	1.28 ± 0.12	1.18 ± 0.26 *	0.84–1.75	1.23 ± 0.18	0.24	−0.16	0.56
	14ND	1.39 ± 0.11	1.21 ± 0.15	0.85–1.63	1.24 ± 0.14	−0.17	−0.13	
	15WH	1.15 ± 0.13	1.05 ± 0.16 *	0.73–1.32	1.01 ± 0.10	0.25	0.08	
	15ER	1.34 ± 0.14	1.11 ± 0.24	0.69–1.69	1.20 ± 0.16	0.06	0.66	
BF	14WH	110.31 ± 21.50	59.68 ± 25.30 ***	10.87–166.95	73.04 ± 30.11	0.63	0.23	0.68
	14ND	141.43 ± 22.30	65.65 ± 32.01 ***	23.15–180.30	84.11 ± 33.22	0.58	0.12	
	15WH	156.48 ± 29.65	82.79 ± 24.56 ***	14.14–227.43	74.21 ± 34.97	1.40	3.24	
	15ER	144.56 ± 31.24	87.95 ± 23.15 ***	10.12–174.03	69.07 ± 30.71	0.96	0.72	
F	14WH	1.33 ± 0.24	0.66 ± 0.32 ***	0.43–1.70	0.97 ± 0.25	0.45	0.15	0.56
	14ND	0.84 ± 0.13	0.59 ± 0.06 ***	0.31–1.10	0.60 ± 0.13	0.30	0.33	
	15WH	1.97 ± 0.56	1.06 ± 0.21 ***	0.44–2.36	1.06 ± 0.31	1.20	3.22	
	15ER	1.38 ± 0.47	1.15 ± 0.54 ***	0.46–1.56	0.87 ± 0.23	0.79	0.34	
SSR	14WH	2.54 ± 1.23	5.86 ± 1.36 ***	1.18–6.80	4.03 ± 1.14	0.19	−0.55	0.62
	15WH	1.87 ± 2.35	8.56 ± 1.54 ***	1.00–9.94	4.02 ± 2.25	0.64	−0.45	

^a^ The significance level between two parents: * *p* ≤ 0.05; ** *p* ≤ 0.01; *** *p* ≤ 0.001.

**Table 2:** Summary of the consensus QTL and their corresponding identified QTL used for QTL meta-analysis.

Trait	Consensus QTL					Identified QTL						
	QTL ^a^	Chr. ^b^	Peak	CI ^c^	PI (kb) ^d^	QTL	LOD ^e^	Peak	CI	Add.	R^2^ (%) ^f^	Env. ^g^
ADL	cqADL.A01-1	A01	25	23.5–25.5	3928–4027	qADL.A01-1	4.02	25	23.5–25.5	0.12	6.87	BLUE
	cqADL.A09-1	A09	48.49	47.43–49.56	5038–6244	qADL.A09-1	6.01	48	47.5–50.5	0.34	14.27	15WH
						qADL.A09-2	3.65	49	47.5–50.5	0.30	9.40	14WH
	cqADL.A09-2	A09	61	60.5–61.5	9254–10,122	qADL.A09-3	3.31	61	60.5–61.5	0.09	5.58	BLUE
	cqADL.C03-1	C03	12.99	10.96–15.03	1378–2930	qADL.C03-1	4.16	13	6.5–13.5	−0.27	9.15	15WH
						qADL.C03-2	5.11	13	8.5–13.5	−0.38	12.77	15ER
	cqADL.C03-2	C03	58	57.5–58.5	21,514–21,931	qADL.C03-3	6.04	58	57.5–58.5	−0.12	10.57	BLUE
	cqADL.C09-1	C09	47	46.5–47.5	32,543–34,424	qADL.C09-1	3.64	47	46.5–47.5	0.31	9.26	14WH
	cqADL.C09-2	C09	102	100.5–102.5	43,321–43,705	qADL.C09-2	5.78	102	100.5–102.5	0.12	10.48	BLUE
	**cqADL.C09-3**	**C09**	**123**	**118.5–123.5**	**45,206–45,833**	**qADL.C09-3**	**5.21**	**123**	**118.5–123.5**	**0.58**	**16.57**	**14ND**
	cqADL.C09-4	C09	129	124.5–129.5	45,832–46,749	qADL.C09-4	3.15	129	124.5–129.5	0.30	7.69	15ER
Cel	cqCel.A08-1	A08	9	7.5–9.5	12,328–13,217	qCel.A08-1	5.08	9	7.5–9.5	−0.26	7.63	BLUE
	cqCel.A10-1	A10	115	113.5–115.5	14,613–14,735	qCel.A10-1	3.35	115	113.5–115.5	−0.21	4.85	BLUE
	cqCel.C02-1	C02	161	154.5–164	43,090–45,788	qCel.C02-1	3.38	161	154.5–164	−0.21	5.13	BLUE
	cqCel.C04-1	C04	0	0–0.5	0–369	qCel.C04-1	3.22	0	0–0.5	−0.69	10.01	15ER
	cqCel.C06-1	C06	67	66.29–67.7	19,870–20,407	qCel.C06-1	4.69	67	66.5–68.5	−0.66	12.09	15WH
						qCel.C06-2	5.95	67	66.5–68.5	−0.31	8.91	BLUE
	cqCel.C06-2	C06	73	72.5–77.5	28,297–33,676	qCel.C06-3	3.68	73	72.5–77.5	−0.52	9.28	14WH
	cqCel.C09-1	C09	118	112.5–118.5	44,583–45,206	qCel.C09-1	3.35	118	112.5–118.5	0.46	8.39	14WH
	cqCel.C09-2	C09	123	118.5–123.5	45,206–45,833	qCel.C09-2	4.82	123	118.5–123.5	0.26	7.21	BLUE
Hem	cqHem.A01-1	A01	13	12.5–13.5	2254–2401	qHem.A01-1	6.73	13	12.5–13.5	−0.28	10.62	BLUE
	cqHem.A03-1	A03	47	45.5–47.5	5031–5777	qHem.A03-1	3.67	47	45.5–47.5	0.38	8.80	14WH
	**cqHem.A07-1**	**A07**	**120**	**119.64–120.35**	**18,917–19,528**	**qHem.A07-1**	**5.26**	**120**	**119.5–120.5**	**−0.50**	**12.96**	**14WH**
						**qHem.A07-2**	**7.84**	**120**	**119.5–120.5**	**−0.24**	**12.82**	**BLUE**
	cqHem.A09-1	A09	55	51.5–59.5	6244–9008	qHem.A09-1	3.73	55	51.5–59.5	−0.15	6.13	BLUE
SG	cqSG.A03-1	A03	45	44.5–45.5	4872–5163	qSG.A03-1	3.77	45	44.5–45.5	−0.04	5.65	14ND
	cqSG.A06-1	A06	34	30.5–34.5	3231–3733	qSG.A06-1	3.54	34	30.5–34.5	0.02	4.40	BLUE
	**cqSG.A07-1**	**A07**	**113**	**112.5–113.5**	**15,704–15,964**	**qSG.A07-1**	**15.06**	**113**	**112.5–113.5**	**0.10**	**27.18**	**14ND**
	cqSG.C01-1	C01	103	98.5–104.5	34,527–36,893	qSG.C01-1	3.73	103	98.5–104.5	0.02	4.64	BLUE
	cqSG.C02-1	C02	34	33.5–34.5	3320–7926	qSG.C02-1	3.24	34	33.5–34.5	−0.05	7.98	14WH
	cqSG.C03-1	C03	4	3.5–6.5	988–1378	qSG.C03-1	5.84	4	3.5–6.5	−0.03	7.51	BLUE
	cqSG.C06-1	C06	69	68.5–69.5	20,407–22,987	qSG.C06-1	9.71	69	68.5–69.5	0.04	12.93	BLUE
	cqSG.C06-2	C06	73	72.5–77.5	28,297–33,676	qSG.C06-2	3.58	73	72.5–77.5	−0.04	5.33	14ND
BF	cqBF.A06-1	A06	129	127.5–129.5	23,209–23,617	qBF.A06-1	3.59	129	127.5–129.5	9.33	8.24	15WH
	cqBF.A07-1	A07	113	111.5–113.5	15,774–15,964	qBF.A07-1	5.13	113	111.5–113.5	12.22	11.97	15WH
	**cqBF.A07-2**	**A07**	**120**	**119.64–120.35**	**18,917–19,528**	**qBF.A07-2**	**6.51**	**120**	**119.5–120.5**	**13.84**	**16.65**	**14WH**
						**qBF.A07-3**	**5.16**	**120**	**119.5–120.5**	**12.96**	**13.13**	**15ER**
	cqBF.A07-3	A07	122	120.5–122	19,528–19,664	qBF.A07-4	6.79	122	120.5–122	5.99	14.34	BLUE
	cqBF.A10-1	A10	134	132.5–134.5	15,279–15,420	qBF.A10-1	3.27	134	132.5–134.5	3.76	6.25	BLUE
	cqBF.C03-1	C03	13	6.5–13.5	1378–2930	qBF.C03-1	3.66	13	6.5–13.5	−3.95	6.89	BLUE
F	cqF.A06-1	A06	34	30.5–34.5	3231–3733	qF.A06-1	3.54	34	30.5–34.5	0.02	4.40	BLUE
	cqF.A06-2	A06	129	128.29–129.7	23,209–23,617	qF.A06-2	3.43	129	127.5–129.5	0.07	8.05	14WH
						qF.A06-3	4.45	129	127.5–129.5	0.08	1.92	15WH
	cqF.A07-1	A07	11	10.5–11.5	1806–1818	qF.A07-1	4.64	11	10.5–11.5	−0.04	7.56	14ND
	cqF.A07-2	A07	52	50.5–53.5	8499–6330	qF.A07-2	5.32	52	50.5–53.5	−0.09	2.34	15WH
	cqF.C01-1	C01	102.7	100.7–104.65	32,292–35,693	qF.C01-1	5.71	102	96.5–103.5	0.09	2.52	15WH
						qF.C01-2	3.98	103	96.5–104.5	0.07	9.39	14WH
						qF.C01-3	3.73	103	98.5–104.5	0.02	4.64	BLUE
	cqF.C03-1	C03	4	2.93–5.06	988–1378	qF.C03-1	4.45	4	3.5–6.5	−0.08	1.92	15WH
						qF.C03-2	5.84	4	3.5–6.5	−0.03	7.51	BLUE
	cqF.C06-1	C06	57	53.5–57.5	15,950–17,544	qF.C06-1	4.83	57	53.5–57.5	0.09	2.13	15WH
	**cqF.C06-2**	**C06**	**69**	**68.71–69.28**	**20,407–22,987**	**qF.C06-2**	**3.26**	**69**	**68.5–69.5**	**0.07**	**7.61**	**14WH**
						**qF.C06-3**	**4.19**	**69**	**68.5–69.5**	**0.08**	**10.76**	**15ER**
						**qF.C06-4**	**9.71**	**69**	**68.5–69.5**	**0.04**	**12.93**	**BLUE**
	cqF.C09-1	C09	21	15.5–21.5	8185–11,082	qF.C09-1	3.68	21	15.5–21.5	−0.07	9.51	15ER
	cqF.C09-2	C09	45	42.5–45.5	30,708–31,732	qF.C09-2	4.06	45	42.5–45.5	−0.04	6.67	14ND
SSR	cqSSR.A03-1	A03	103	101.5–103.5	17,026–17,802	qSSR.A03-1	3.33	103	101.5–103.5	−0.15	5.43	BLUE
	cqSSR.A05-1	A05	83	80.5–84.5	20,644–20,666	qSSR.A05-1	4.56	83	80.5–84.5	0.28	7.04	14WH
	cqSSR.A09-1	A09	149	148.5–149	32,939–33,464	qSSR.A09-1	4.37	149	148.5–149	0.17	7.18	BLUE
	cqSSR.C01-1	C01	80	73.5–80.5	13,067–28,388	qSSR.C01-1	5.65	80	73.5–80.5	−0.31	8.77	14WH
	cqSSR.C02-1	C02	65	53.5–65.5	1896–5009	qSSR.C02-1	3.64	65	53.5–65.5	−0.17	5.97	BLUE
	cqSSR.C06-1	C06	58	57.5–58.5	17,544–17,599	qSSR.C06-1	3.24	58	57.5–58.5	−0.26	4.91	14WH
	cqSSR.C07-1	C07	50	49.5–50.5	33,958–34,672	qSSR.C07-1	3.84	50	49.5–50.5	0.16	6.39	BLUE
	cqSSR.C07-2	C07	82	81.5–82.5	40,805–40,986	qSSR.C07-2	3.72	82	81.5–82.5	0.25	5.63	14WH
	**cqSSR.C09-1**	**C09**	**118**	**116.79–119.19**	**44,590–45,206**	**qSSR.C09-1**	**7.40**	**118**	**114.5–118.5**	**0.23**	**12.64**	**BLUE**
						**qSSR.C09-2**	**5.05**	**118**	**115.5–118.5**	**0.65**	**14.26**	**15WH**
	**cqSSR.C09-2**	**C09**	**122**	**119.5–123.5**	**45,206–45,833**	**qSSR.C09-3**	**9.93**	**122**	**119.5–123.5**	**0.44**	**17.11**	**14WH**

^a^ Consensus QTL name. The major consensus QTL are in bold font. ^b^ Chromosome. ^c^ Confidence interval. ^d^ Physical interval. ^e^ Logarithm of odds. ^f^ Phenotypic variation explained by additive effect. ^g^ Environment.

**Table 3:** Unique QTL information involved in more than two traits in this study.

Unique QTL				Consensus QTL			
	Chr. ^a^	Peak	CI ^b^		Trait	Peak	CI
uqA06-1	A06	34	32.58–35.41	cqSG.A06-1	SG	34	30.5–34.5
				cqF.A06-1	F	34	30.5–34.5
uqA06-2	A06	129	128.42–129.57	cqBF.A06-1	BF	129	127.5–129.5
				cqF.A06-2	F	129	128.29–129.7
uqA07-1	A07	113	112.55–113.44	cqBF.A07-1	BF	113	111.5–113.5
				cqSG.A07-1	SG	113	112.5–113.5
uqA07-2	A07	120	119.74–120.25	cqHem.A07-1	Hem	120	119.64–120.35
				cqBF.A07-2	BF	120	119.64–120.35
uqC01-1	C01	102.77	101.12–104.42	cqSG.C01-1	SG	103	98.5–104.5
				cqF.C01-1	F	102.68	100.7–104.65
uqC03-1	C03	4	3.13–4.86	cqF.C03-1	F	4	2.93–5.06
				cqSG.C03-1	SG	4	3.5–6.5
uqC03-2	C03	12.99	11.23–14.75	cqBF.C03-1	BF	13	6.5–13.5
				cqADL.C03-1	ADL	12.99	10.96–15.03
uqC06-1	C06	69	68.75–69.24	cqSG.C06-1	SG	69	68.5–69.5
				cqF.C06-2	F	69	68.71–69.28
uqC06-2	C06	73	71.23–74.76	cqCel.C06-2	Cel	73	72.5–77.5
				cqSG.C06-2	SG	73	72.5–77.5
uqC09-1	C09	118	116.88–119.11	cqCel.C09-1	Cel	118	112.5–118.5
				cqSSR.C09-1	SSR	118	116.79–119.19
uqC09-2	C09	122.56	121.23–123.88	cqADL.C09-3	ADL	123	118.5–123.5
				cqCel.C09-2	Cel	123	118.5–123.5
				cqSSR.C09-2	SSR	122	119.5–123.5

^a^ Chromosome. ^b^ Confidence interval.

**Table 4:** Summary of the combined QTL detected by QEI mapping.

Trait	Combined QTL ^a^	Chr. ^b^	Pos. ^c^	CI ^d^	LOD ^e^	LOD	LOD	PVE ^h^	PVE	PVE	Add ^k^	Consensus QTL ^l^
						(A) ^f^	(AbyE) ^g^		(A) ^i^	(AbyE) ^j^		
ADL	IqADL.A09-1	A09	51	50.5–51.5	8.19	3.86	4.32	6.26	3.62	2.64	0.13	
	IqADL.C03-1	C03	13	9.5–13.5	8.53	5.08	3.44	7.05	4.85	2.20	−0.15	cqADL.C03-1
	IqADL.C03-2	C03	58	57.5–58.5	5.11	3.99	1.12	4.57	3.77	0.80	−0.13	cqADL.C03-2
	**IqADL.C09-1**	**C09**	**123**	**118.5–123.5**	**6.16**	**2.35**	**3.81**	**8.56**	**2.25**	**6.32**	**0.10**	**cqADL.C09-3**
Cel	IqCel.C02-1	C02	162	156.5–164	6.65	5.13	1.52	2.49	1.51	0.98	−0.24	cqCel.C02-1
	IqCel.C06-1	C06	67	66.5–68.5	5.63	3.18	2.44	1.87	1.00	0.87	−0.22	cqCel.C06-1
	IqCel.C07-1	C07	81	80.5–81.5	57.23	26.20	31.03	24.29	8.49	15.79	0.58	
	IqCel.C07-2	C07	83	82.5–83.5	48.65	8.31	40.35	18.45	2.58	15.87	−0.32	
	IqCel.C09-1	C09	118	116.5–118.5	8.53	7.72	0.81	2.66	2.37	0.29	0.31	cqCel.C09-1
Hem	IqHem.A01-1	A01	15	14.5–15.5	6.63	6.01	0.62	3.42	3.36	0.06	−0.26	
	IqHem.A03-1	A03	47	45.5–47.5	5.77	4.41	1.36	3.15	2.47	0.68	0.16	cqHem.A03-1
	**IqHem.A07-1**	**A07**	**120**	**119.5–120.5**	**10.11**	**9.28**	**0.84**	**5.76**	**5.26**	**0.50**	**−0.25**	**cqHem.A07-1**
SG	IqSG.A07-1	A07	116	115.5–117.5	13.87	2.23	11.63	22.34	4.16	18.18	0.01	
BF	**IqBF.A07-1**	**A07**	**120**	**119.5–120.5**	**9.72**	**4.59**	**5.13**	**10.09**	**5.43**	**4.65**	**4.34**	**cqBF.A07-2**
	IqBF.C03-1	C03	13	6.5–13.5	5.22	4.86	0.37	7.04	6.08	0.96	−4.31	cqBF.C03-1
F	**IqF.C06-1**	**C06**	**69**	**68.5–69.5**	**40.70**	**18.15**	**22.55**	**23.75**	**9.61**	**14.15**	**0.07**	**cqF.C06-2**
	IqF.C06-2	C06	72	71.5–72.5	26.87	2.32	24.55	13.36	1.18	12.18	−0.02	
	IqF.C09-1	C09	21	16.5–21.5	6.62	4.94	1.68	2.80	2.45	0.35	−0.03	cqF.C09-1
SSR	IqSSR.A05-1	A05	83	80.5–84.5	4.96	0.59	4.38	5.72	2.04	3.68	0.12	cqSSR.A05-1
	IqSSR.C01-1	C01	80	74.5–80.5	5.48	2.13	3.35	7.40	6.70	0.70	−0.21	cqSSR.C01-1
	IqSSR.C06-1	C06	63	61.5–65.5	4.02	1.33	2.69	5.08	4.31	0.78	−0.19	
	**IqSSR.C09-1**	**C09**	**122**	**119.5–123.5**	**10.30**	**2.08**	**8.22**	**11.94**	**6.40**	**5.54**	**0.21**	**cqSSR.C09-2**

^a^ Combined QTL is the QTL detected by QEI mapping with ICIM algorithm. The corresponding major consensus QTL detected by CIM algorithm are in bold font. ^b^ Chromosome. ^c^ Chromosomal position (cM) of the peak. ^d^ Confidence interval. ^e^ LOD sore for additive and QEI effect. ^f^ LOD score for additive effect. ^g^ LOD score for QEI effect. ^h^ Phenotypic variation explained by additive and QEI effect. ^i^ Phenotypic variation explained by additive effect. ^j^ Phenotypic variation explained by QEI effect. ^k^ Estimated average additive effect of the QTL. ^l^ The corresponding major consensus QTL detected by CIM algorithm.

## Data Availability

Not applicable.

## Supplementary Materials

The following supporting information can be downloaded at: https://www.mdpi.com/article/10.3390/plants11030373/s1, Table S1: The broad-sense heritabilities of ADL, Cel, Hem, SG, BF, F, and SSR in this study, Table S2: Correlations between ADL, Cel, Hem, SG, BF, F, and SSR, based on the BLUE data, Table S3: List of the candidate genes in this study, Table S4: Comparative analysis of the QTL identified in the present and previous studies based on the *B. napus* genome sequences, Table S5: Summary of the environments where the parents and DH population were grown.
